# A Bi-Dimensional Taxonomy of Social Responsivity in Middle Childhood: Prosociality and Reactive Aggression Predict Externalizing Behavior Over Time

**DOI:** 10.3389/fpsyg.2020.586633

**Published:** 2021-01-15

**Authors:** Simone Dobbelaar, Anna C. K. van Duijvenvoorde, Michelle Achterberg, Mara van der Meulen, Eveline A. Crone

**Affiliations:** ^1^Leiden Consortium on Individual Development, Leiden University, Leiden, Netherlands; ^2^Developmental and Educational Psychology, Faculty of Social and Behavioural Sciences, Leiden University, Leiden, Netherlands; ^3^Leiden Institute for Brain and Cognition, Leiden, Netherlands; ^4^Department of Psychology, Education and Child Studies, Erasmus School of Social and Behavioural Sciences, Erasmus University Rotterdam, Rotterdam, Netherlands

**Keywords:** prosociality, reactive aggression, externalizing behavior, internalizing behavior, middle childhood

## Abstract

Developing social skills is essential to succeed in social relations. Two important social constructs in middle childhood, prosocial behavior and reactive aggression, are often regarded as separate behaviors with opposing developmental outcomes. However, there is increasing evidence for the co-occurrence of prosociality and aggression, as both might indicate responsivity to the social environment. Here, we tested whether a bi-dimensional taxonomy of prosociality and reactive aggression could predict internalizing and externalizing problems over time. We re-analyzed data of two well-validated experimental tasks for prosociality (the Prosocial Cyberball Game) and reactive aggression (the Social Network Aggression Task) in a developmental population sample (*n* = 496, 7–9 years old). Results revealed no associations between prosociality and reactive aggression, confirming the independence of those constructs. Interestingly, although prosociality and reactive aggression independently did not predict problem behavior, the interaction of both was negatively predictive of changes in externalizing problems over time. Specifically, only children who scored low on both prosociality and reactive aggression showed an increase in externalizing problems 1 year later, whereas levels of externalizing problems did not change for children who scored high on both types of behavior. Thus, our results suggest that at an individual level, reactive aggression in middle childhood might not always be maladaptive when combined with prosocial behavior, thereby confirming the importance of studying social competence across multiple dimensions.

## Introduction

One of the major developmental tasks that children face during childhood is to develop skills that help them to respond adequately to changes in their social environment. Social skills can ensure that children succeed in their social relations (Newcomb et al., 1993). In particular, middle childhood seems to be an important phase to study the development of social competence. In this phase, children spend an increasing amount of time at school with peers and start to form dyadic friendships based on shared interests (Berndt, 2004). Thus, middle childhood is marked by an expansion of children’s social world in which social skills are important for social adjustment (Mchale et al., 2003; Del Giudice et al., 2009). Problems in the development of social behavior, for example being unable to develop close friendships and gain social acceptance, can result in risk for psychological and behavioral difficulties (Burt et al., 2008; Bornstein et al., 2010).

Prior research demonstrated an important role of prosociality and aggression in predicting developmental outcomes later in life. Prosociality, defined as voluntary behaviors benefiting others (Eisenberg and Mussen, 1989), has consistently been associated with positive psychosocial outcomes and decreases in externalizing and internalizing problems (Memmott-Elison et al., 2020). On the other hand, aggressive behavior has often been associated with detrimental outcomes. In particular, reactive aggression, the defensive or retaliatory response to provocation and frustration (Crick and Dodge, 1996), has been related to cases of externalizing problems, such as emotional dysregulation and hyperactivity symptoms, as well as internalizing problems (Card and Little, 2006; Mcauliffe et al., 2006; Mathieson and Crick, 2010; White et al., 2013). As from here, we define reactive aggression as the self-protective response following social rejection. Given these opposing relations to developmental outcomes, reactive aggression and prosociality are often considered opposing constructs that are negatively related (Card and Little, 2006).

Recently, it was argued that treating prosociality and reactive aggression as opposing relations might be too limited for capturing the dynamics of these developmental relations (Crone et al., 2020). An alternative way to address this is by using bi-dimensional models, in which the intersection of two constructs results in four different “quadrants” of behavioral profiles. This approach has been used successfully before, for example to categorize responses to rejection on an antisocial-prosocial and engaged-disengaged dimension (Sunami et al., 2019) or to identify factors that can explain why some adolescents are both prosocial as well as rebellious (Blankenstein et al., 2019). Here, we propose a bi-dimensional taxonomy of social responsivity to examine the relation between prosociality and reactive aggression (Figure 2; see also Crone et al., 2020), based on the idea that both prosociality and reactive aggression indicate responsiveness to changes in the social environment (i.e., responsivity to rejection of others and self, respectively). It was previously suggested that reactive aggression may result specifically from threats to self-evaluation (Yoon et al., 2018), whereas prosociality may foster positive self-evaluation (Crone and Fuligni, 2020).

Indeed, previous studies in adolescents support the idea that prosociality and aggression can co-occur in individuals, and that this combination can result in positive psychosocial outcomes. For instance, Hawley (1999, 2003) showed that adolescents who were prosocial as well as aggressive were among the most socially dominant, socially skilled and liked by peers, compared to the adolescents that used only one or none of the strategies. Despite their aggressive strategies, their friendships were rated as intimate and fun (Hawley et al., 2007). The resource control theory (Hawley, 1999) proposes that both prosocial as well as aggressive strategies can be used to achieve social goals and status. Individuals that use both strategies (also labeled “bi-directional controllers”) might be the ones who are most responsive to their environment, since they seem to be able to successfully adapt their behavior based on the social goal they try to achieve (Hawley, 2003). On the other hand, children that used neither prosocial nor aggressive strategies (i.e., the “non-controllers”) showed the least attention to social cues (Hawley, 2003), which might indicate a lack of adaptation to the social environment. These children were also the least popular and most peer neglected and rejected (Hawley, 2003), which could suggest being more prone to developing psychosocial problems.

Taken together, two essential types of social competence behavior that are rapidly developing during middle childhood are prosociality (Van Der Meulen et al., 2018) and the regulation of aggression (Achterberg et al., 2018, 2020). These two constructs are often studied independently, even though the combination may be more predictive for developmental outcomes. Whether the interaction of prosociality and reactive aggression might be a better predictor of problem behavior is currently unknown. We hypothesize that reactive aggression in combination with prosociality may buffer against disadvantageous developmental outcomes, based on prior research showing that popular adolescents who are aggressive (Rodkin et al., 2000; De Bruyn and Cillessen, 2006) show prosocial behavior as well (Lafontana and Cillessen, 2002; Cillessen and Rose, 2005). Studies investigating the relation with psychosocial adjustment, however, mainly focused on the antisocial aspect of this group (Sandstrom and Cillessen, 2006; Rose and Swenson, 2009). It should further be noted that these studies mainly focused on proactive aggression, the more goal-directed and deliberate form of aggression (Crick and Dodge, 1996). As both proactive and reactive aggression indicate a certain responsiveness to changing social contexts, either to achieve social goals or to defend oneself or others, we would expect similar relations. Reactive aggression is often found to be related to more behavioral problems than proactive aggression (Card and Little, 2006), but it seems to have less negative outcomes when it is investigated as a self-protective response instead of a bias to overattribute hostility to others (Pulkkinen, 1996). However, to our knowledge, the co-occurrence of prosociality and reactive aggression has not been studied before. Thus, the present study examined whether prosociality, reactive aggression or a combination of both predicts problem behavior during middle childhood, in a longitudinal population sample.

That is, we tested the bi-dimensional taxonomy of prosociality and reactive aggression in the Leiden Consortium on Individual Development (L-CID). In this longitudinal twin study, prosociality and reactive aggression were measured in a large sample (*n* = 496, 7–9 years old) with two well-validated tasks, the Prosocial Cyberball Game (PCG; Vrijhof et al., 2016; Van Der Meulen et al., 2017) and the Social Network Aggression Task (SNAT; Achterberg et al., 2017). First, we tested whether there was a negative association between prosociality and reactive aggression (Card and Little, 2006), a positive association (Hawley, 2003; Cillessen and Rose, 2005), or no association at all. Second, we tested whether individual differences in the relation between prosociality and reactive aggression could predict internalizing and externalizing problems, both cross-sectionally as well as 1 year later. We expected that prosociality would negatively predict both internalizing as well as externalizing problems (Memmott-Elison et al., 2020), whereas we expected reactive aggression to be positively related to both internalizing and externalizing problems (Card and Little, 2006). However, based on our bi-dimensional taxonomy, we hypothesized that reactive aggression in combination with prosociality might serve as buffer against both internalizing and externalizing problems, as it indicates the most adaptation to their social environment, whereas children who lack both types of behavior might be more vulnerable to developing problem behavior.

## Materials and Methods

### Participants

This study was part of the larger longitudinal twin study of the L-CID, that focuses on the development of social competence and behavioral control and aims to unravel why not all children are equally responsive to variations in their (social) environment (Euser et al., 2016; Crone et al., 2020). We reanalyzed and extended data previously reported by Achterberg et al. (2018) and Van Der Meulen et al. (2018). Families with same-sex twins born between 2006 and 2009 that lived in the Western municipalities of the Netherlands were invited to participate. Address information of these families was obtained from municipalities registries. Participants were included when they were fluent in Dutch and had normal or corrected to normal vision. The study was approved by the Dutch Central Committee on Research Involving Human Subjects (CCMO) and informed consent was obtained from both parents. The data included in this study were collected in 2015–2016 (Time Point 1: T1) and 1 year later, in 2016–2017 (Time Point 2: T2).

At T1, 512 participants (of 256 families) were included. Of these participants, 11 were diagnosed with an Axis-I disorder: nine with attention deficit hyperactivity disorder (ADHD) and/or attention deficit disorder (ADD), one with generalized anxiety disorder (GAD) and one with pervasive developmental disorder not otherwise specified (PDD-NOS). Because the aim was to represent a population sample, all participants were included in the study. Exclusion criteria were incomplete data: at T1, three participants had incomplete data from the SNAT due to technical problems and 13 participants did not complete the PCG, due to technical errors (*n* = 2) and due to early termination of the MRI procedure (*n* = 11, due to, e.g., anxiety or falling asleep), since both tasks were administered in an MRI scanner. Therefore, our final sample for testing the association between prosociality and reactive aggression consisted of 496 participants [mean age: 7.95 ± 0.67, 52.2% female, socioeconomic status (SES): 9% low, 45% middle, 46% high]. Internalizing and externalizing problem behavior was studied using parental reports of the Strengths and Difficulties Questionnaire (SDQ). Twenty-four participants did not have any SDQ data at T1, and were therefore excluded from further analyses. Of the resulting participants, SDQ data of 451 participants were collected at T2. Thus, the final sample for testing the predictive value of the bi-dimensional taxonomy for problem behavior consisted of 451 participants (91% of the sample, mean age: 7.95 ± 0.67, 52.8% female, SES: 9% low, 45% middle, 46% high). Demographic characteristics are presented in Table 1. Socio-economic status was based on parental education and calculated as follows: high SES included families in which both parents received at least preparatory college education. Low SES included families where both parents’ completed at most vocational education. The remaining combinations were included in the middle SES category.

**TABLE 1:** Demographic characteristics.

	Bi-dimensional	Longitudinal associations
	taxonomy (T1)	(T1 and T2)
*N*	496	451
Female	259 (52.2%)	238 (52.8%)
Age (SD) at T1	7.95 (0.67)	7.95 (0.67)
Age range	7.02–9.68	7.02–9.68
AXIS-I disorder at T1	11 (2.2%)^1^	9 (2%)^2^
IQ (SD) at T1	103.77 (11.72)	104.07 (11.60)
IQ range	72.50–137.50	72.50–137.50
SES low–middle–high^3^ at T1	9–45–46%	9–45–46%

*^1^9 ADHD and/or ADD; 1 PDD-NOS, 1 GAD. ^2^7 ADHD and/or ADD; 1 PDD-NOS, 1 GAD. ^3^Socioeconomic status, based on parental education.*

### Procedure

At T1, participants and their primary caregiver (i.e., the parent who, according to self-report, spent most time with the children) were invited to the Leiden University Medical Center (LUMC) to participate in a behavioral and MRI session. Participants received instructions on how to perform the SNAT and PCG and practiced the tasks on a laptop. The tasks were completed in the MRI scanner, but for the purpose of this study, only the behavioral results were analyzed. Since the study was part of the larger L-CID study, other behavioral and parent-child interaction tasks were performed as well (Crone et al., 2020). During the visit, one child participated in the MRI session, while the other performed the additional behavioral tasks. Within a twin pair, it was randomly assigned whether the oldest or youngest started with the MRI session or the additional behavioral tasks. After completion of a 1-h scan session, participants answered exit questions on both tasks outside of the scanner. At T2, participants were visited at home. In both years, both parents were asked to fill out questionnaires online before the day of the visit.

### Measures

#### Social Network Aggression Task

**FIGURE 1:**
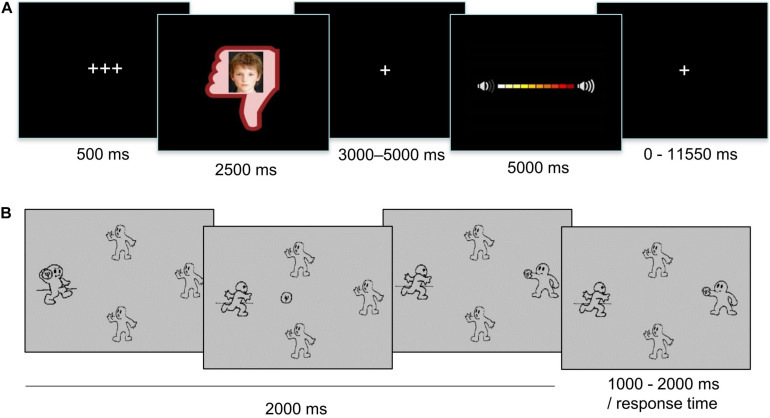
Schematic representations of a trial in **(A)** the Social Network Aggression Task and **(B)** the Prosocial Cyberball Game.

To measure reactive aggression, the SNAT was used. This task was previously described and validated as a reliable measure of rejection-related aggression (Achterberg et al., 2016, 2017, 2018). Prior to the lab visit, participants filled out a personal profile with questions about their favorite food or sports, and sent this back at least a week before the lab visit. During the lab visit, participants were told that unknown, same-aged peers had judged their profile and had provided feedback on whether they liked their profile (positive feedback), disliked their profile (negative feedback) or did not know whether they liked it or not (neutral feedback). Next, participants performed the actual task in the MRI scanner, where they were presented with pictures of these peers in either a green thumb up (positive feedback), a red thumb down (negative feedback) or a gray circle (neutral feedback). Following this feedback, participants had to imagine they could send a noise blast to the peer that judged them by pressing a button with their right index finger. The noise blast would increase in sound as they continued to press the button for a longer duration. The specific instruction to imagine was used to reduce the amount of deception used in the task. Previous research showed that imagery can also result in aggressive reactions (Konijn et al., 2007). Participants were instructed to always press the button, but to choose the duration of the button press themselves. The duration of the button press (and thus the volume of the sound blast) was displayed in a volume bar (Figure 1A). During the practice session of the task, the sound of the volume bar was presented twice: once with increasing volume for each colored block, and once with the maximum volume. Participants were informed that they would not hear the sound during the task, but that they should merely imagine sending the sound to the other peer. Subsequently, they practiced six trials of the task (with each feedback condition presented twice). Unknown to the participants, the peers shown on the photographs were not real children. Every picture consisted of two morphed photographs from an existing database. The photographs were randomly matched to the valence of the feedback, in such a way that every picture was simultaneously presented with either positive, neutral or negative feedback. Because the SNAT would be administered again at later time points in the longitudinal study, participants were not debriefed about the deception on the day of the lab visit. Debriefing will take place at the final phase of the L-CID study (Crone et al., 2020).

**FIGURE 2:**
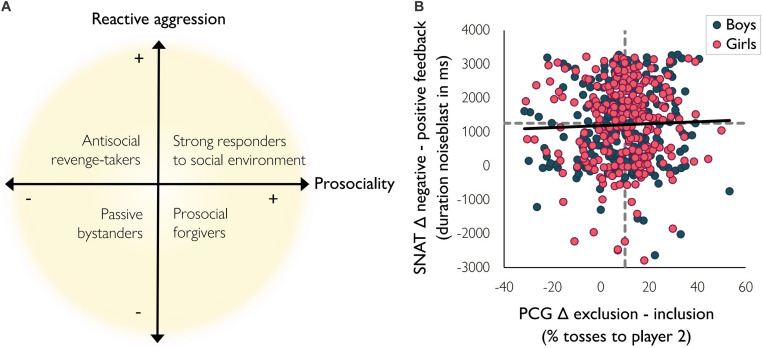
**(A)** Bi-dimensional model of prosociality and reactive aggression as proposed by Crone et al. (2020). **(B)** Relation between prosociality (difference in PCG percentage tosses to player 2 in the exclusion blocks and inclusion block) and reactive aggression (difference in SNAT duration noise blast after negative trials and positive trials). A higher PCG difference score indicates more prosociality; a higher SNAT difference score indicates more reactive aggression. Dotted lines represent median scores.

The SNAT consisted of 60 trials: three blocks of 20 trials, with 20 trials in total for each feedback condition (positive, negative, and neutral). The order of presentation of trials was pseudo-randomized, to ensure that no more than three trials from the same feedback condition were presented consecutively. Each trial started with a fixation screen of 500 ms, after which the social feedback screen was presented for 2500 ms. Next, a jittered fixation screen appeared for 3000–5000 ms, followed by the noise screen displaying the volume bar for 5000 ms. Participants were instructed to press the button as fast as they could to send a noise blast to the peer. When participants did not respond within 1500 ms, a screen with the text “*too late!*” was presented for the remaining 3500 ms. If they did press the button in time, a new colored box (ranging from yellow to red) would appear on the volume bar each 350 ms, indicating the volume of the noise blast. When participants released the button, or after 3500 ms, no more colored boxes were added and the volume bar was presented for the remaining of the 5000 ms. The trial ended with an intra-trial fixation screen with a jitter of 0–11,550 ms.

After completion of the SNAT, an exit interview was administered to check whether the social feedback manipulation worked. Participants answered questions on how much they liked the task in general, the feedback in each feedback condition (e.g., “*How much did you like reactions with a green thumb up?*”) and the fact that they could send a noise blast. They could answer the questions on a 6-point scale, ranging from “*very little*” (1) to “*very much*” (6). As reported in Achterberg et al. (2018), the social feedback manipulation was successful: on average, participants liked negative feedback significantly less than neutral and positive feedback, and they liked positive feedback the most.

#### Prosocial Cyberball Game

To measure prosociality, an fMRI adapted version of the PCG was used. This paradigm has previously been validated as a measure of prosocial compensating behavior (Van Der Meulen et al., 2016, 2017, 2018; Vrijhof et al., 2016). The PCG is a virtual ball tossing game. In the task, four players are presented on the screen: one illustrates the participant (at the bottom of the screen), the other three represent the other three players (Figure 1B). Participants had to toss a ball to one of the three other players by pressing a button. They were instructed to imagine playing the game in a social setting, for example by imagining what the other three players looked like or in what kind of place they were playing the game. Previous research showed that imagining playing a game with others led to the same results as when other players were actually present (Zadro et al., 2004). The task consisted of three blocks. The first block was an “inclusion block,” in which each participant received the ball an equal amount of times (25% for each player). Critically, in the “exclusion blocks” (block 2 and 3) player 2 (at the top of the screen) was excluded by players 1 and 3, such that he did not receive the ball from these two players anymore. However, player 2 still tossed the ball to each player an equal amount of times. Thus, in trials where player 1 or 3 tossed the ball, the participant received it 50% of the trials, whereas in trials where player 2 tossed the ball, the participant received it 33% of the trials.

The inclusion block (120 trials) was administered on a laptop outside of the MRI scanner. The exclusion blocks (168 trials in total, 84 trials per block) were performed in the MRI scanner. Each trial consisted of a ball toss and lasted 2000 ms. Intra-trial intervals were jittered from 1000–2000 ms. For trials in which the participant was tossing the ball, the response time of the participant represented the jitter. Participants were instructed to toss the ball by pressing a button with a finger on their right hand (index finger, middle finger, or ring finger for player 1, 2, and 3, respectively).

Again, after completing the PCG, an exit interview was administered to check whether participants felt differently toward the excluded player and the excluding players. Participants answered questions on how much they liked each player (e.g., “*How much did you like player 1?*”) on a 6-point scale ranging from “*very little*” (1) to “*very much*” (6). They also indicated to which player they would like to donate a sticker (“*If you could donate a sticker to one of the three players, which one would you choose?*”). As reported in Van Der Meulen et al. (2018), the exit questions confirmed that participants liked the excluded player more than the excluding players, and that likeability of the excluding players did not differ. In addition, the majority of participants indicated they would donate the sticker to the excluded player.

#### Strengths and Difficulties Questionnaire

To measure internalizing and externalizing problem behavior, we used the SDQ (Goodman, 2001). The SDQ measures psychosocial problems in children of 4–17 years old, and was completed by both parents. In the study, we differentiated between the primary caregiver that spent most time with the child at the start of the study (“primary parent,” PP), and the other caregiver (“other parent,” OP). However, since often the OP spent an equal amount of time with their children as the PP or even started spending more time than the PP over time, we combined the reports of both parents for a more reliable measure of problem behavior. Specifically, we used four subscales of the SDQ: Emotional Problems (e.g., “*My child worries a lot*,” PP: α_T__1_ = 0.70, α_T__2_ = 0.76; OP: α_T__1_ = 0.69, α_T__2_ = 0.74), Peer Problems (e.g., “*My child is picked on or bullied by other children*,” PP: α_T__1_ = 0.51, α_T__2_ = 0.54; OP: α_T__1_ = 0.59, α_T__2_ = 0.51), Hyperactivity (e.g., *“My child is restless, overactive, cannot stay still for long*,” PP: α_T__1_ = 0.82, α_T__2_ = 0.81; OP: α_T__1_ = 0.76, α_T__2_ = 0.80), and Conduct Problems (e.g., *“My child often has temper tantrums or hot tempers*,” PP: α_T__1_ = 0.59, α_T__2_ = 0.57; OP: α_T__1_ = 0.53, α_T__2_ = 0.55). Each subscale consisted of five items that were answered on a three-point Likert scale (0 = not true, 1 = somewhat true, 2 = certainly true). Prior research proposed combining the Emotional Problems and Peer Problems subscales into an Internalizing scale, and the Hyperactivity and Conduct Problems subscales into an Externalizing scale. These two broader subscales might be more advantageous to use in low-risk samples, whereas the use of the subscales separately is encouraged when screening for disorders (Goodman et al., 2010). Because we aimed to look at a more general form of problem behavior and our sample had a relatively low amount of clinical disorders, we decided to use the Internalizing (PP: α_T__1_ = 0.72, α_T__2_ = 0.75; OP: α_T__1_ = 0.70, α_T__2_ = 0.72) and Externalizing (PP: α_T__1_ = 0.78, α_T__2_ = 0.79; OP: α_T__1_ = 0.76, α_T__2_ = 0.78) subscales.

First, we recoded items in the Conduct Problems, Hyperactivity, and Peer Problems subscales, such that higher scores indicated more problems/hyperactivity. Subscales were calculated as the total score of the five items. Following the scoring algorithms of the SDQ,^1^ incomplete subscale scores were prorated to a five-item scale if at least three items per subscale were present. If not, data for that subscale was defined as missing and left out of the analysis. Subsequently, the Internalizing and Externalizing subscales were calculated by summing the total score of the Emotional Problems and Peer Problems and the total score of Hyperactivity and Conduct Problems, respectively. The correlations between the ratings of both parents on each subscale were significant (Internalizing: T1: *r* = 0.62; T2: *r* = 0.63; Externalizing: T1: *r* = 0.66; T2: *r* = 0.71; all *p’*s < 0.001). Thus, we averaged the Internalizing and Externalizing scores for both parents on each time point and proceeded with these variables in the subsequent analyses. For 126 participants only one parent had complete SDQ scores on one or both time points. To include as many participants as possible, we included these participants in the analysis with the SDQ score of one parent. For significant results, we performed additional sensitivity analyses where we checked whether the results changed if we excluded participants with solo-parental report (vs multi-parental reports).

### Data Analysis

We defined reactive aggression as the difference score in mean reaction time (in ms) of trials in the negative feedback condition and trials in the positive feedback condition of the SNAT. Prosocial compensating behavior was defined as the mean percentage tosses to player two in the exclusion blocks subtracted by the percentage tosses to player two in the inclusion block of the PCG. Theoretically, combining the two dimensions resulted in four different quadrants (see Figure 2A): scoring low on prosociality as well as on reactive aggression can be defined as the “passive bystanders,” who do not differentiate their behavior based on the social context (lower left quadrant). Individuals scoring low on prosociality but high on reactive aggression can be defined as the “antisocial revenge-takers” (upper left quadrant). Scoring high on prosociality but low on reactive aggression are the individuals that can be labeled as “prosocial forgivers” (lower right quadrant). Finally, individuals who show prosocial behavior as well as reactive aggression might be “strong responders to the social environment,” as they change their behavior based on the social context (upper right quadrant). For our analyses, however, we investigated reactive aggression and prosocial behavior on a continuous scale to optimally use variation in these constructs.

First, to investigate the relation between prosocial compensating behavior and reactive aggression, we ran a bivariate correlation on the two variables. Second, we performed regression analyses to test whether prosociality and reactive aggression were independently (i.e., corrected for each other) related to internalizing and externalizing problems at the same time point (T1). In addition, we created an interaction variable of prosociality and reactive aggression to test specific quadrants of the bi-dimensional taxonomy in a separate regression analysis. Specifically, we performed this analysis without main effects to test whether the combination of high levels of prosociality and reactive aggression (i.e., the quadrant of the “strong responders to social environment”) was related to internalizing or externalizing problems at T1 (see Blankenstein et al., 2019, for a similar approach). Third, in longitudinal regression analyses, we tested whether prosociality and reactive aggression were predictive for internalizing or externalizing problems 1 year later (T2), corrected for the level of problems at T1. Again, to test our bi-dimensional taxonomy, we also tested whether the interaction variables could predict these problems at T2. Predictor variables in each regression analysis were transformed to *z*-scores, to be able to compare regression coefficients. Data points with *z*-values below −3.29 or above 3.29 were defined as outliers and were winsorized (Tabachnick and Fidell, 2013). Because twins are nested within families, the data violated the assumption of homoscedasticity. To correct for this violation, we used heteroscedasticity-consistent standard errors (HCSE) estimators (Hayes and Cai, 2007) in all analyses.

Following Blankenstein et al. (2019), the interaction variable of prosociality and reactive aggression was created as follows: first, the SNAT and PCG score were transformed to *z*-values. Next, a constant was added to the *z*-values, to make all values positive, before multiplying both terms. This created an interaction variable in which high scores were indicative of the upper-right quadrant of the model (“strong responders to social environment”: high prosociality, high reactive aggression) and low scores indicated the lower-left quadrant (“passive bystanders”: low prosociality, low reactive aggression).

For each regression analysis, we checked the statistical assumptions of normality of residuals (by inspecting histograms and P–P plots), the absence of multicollinearity [Variance Inflation Factor (VIF) < 10] and the assumption of homoscedasticity. In the cross-sectional analyses, the residuals followed a positively skewed distribution. However, using a square root transformation on the dependent variables, we obtained similar results. Therefore, we report the results of the data without transformation for better interpretation. In the longitudinal analyses, the residuals were normally distributed. There was no evidence of multicollinearity in any of the regression analyses (all VIF < 1.1). To control for multiple comparisons, we used a Bonferroni procedure for correlated comparisons.^2^ In this procedure, the correlation between outcome variables is taken into account when controlling for multiple tests with multiple outcome variables. The average correlation between internalizing T1, internalizing T2, externalizing T1 and externalizing T2 was *r* = 0.43, yielding a significance level of α = 0.029 for four test outcomes.

## Results

### Relation Prosociality and Reactive Aggression

Average prosociality and reactive aggression were not significantly correlated, *r* = 0.05, *p* = 0.34. To check whether the difference score specifically drove this absence of effect, we also calculated the correlations for the separate variables (i.e., the noise blast duration after negative feedback and the noise blast duration after positive feedback in the SNAT, and the mean percentage tosses to player two in the excluding blocks and the percentage tosses to player two in the including block in the PCG). These analyses confirmed that there was no correlation between the SNAT and PCG variables (all *p’*s > 0.05, see Supplementary Table S1). Together, these findings suggest that prosociality and reactive aggression are separable constructs. Nevertheless, substantial individual variation was noted in the association between prosociality and reactive aggression (Figure 2B).

### Cross-Sectional Predictions of Internalizing and Externalizing Behavior

Next, we tested whether prosociality (difference in percentage tosses to player 2 in exclusion blocks and inclusion block of the PCG) and reactive aggression (difference noise blast duration after negative and positive feedback in the SNAT), and its interaction term were correlated with problem behavior, by analyzing all measures at T1, using multiple regression analyses.

First, we ran two multiple regression analyses with prosociality and reactive aggression as predictors for internalizing and externalizing problems, respectively. Results showed that prosociality and reactive aggression did not predict internalizing problems [*R*^2^ = 0.004, *F*(2,448) = 0.93, *p* = 0.40], nor externalizing problems [*R*^2^ = 0.004, *F*(2,448) = 0.86, *p* = 0.42] at the same time point. A separate single regression analysis for the interaction term of prosociality and reactive aggression (prosociality^∗^reactive aggression) as predictor also revealed no relation with internalizing problems [*R*^2^ = 0.005, *F*(1,449) = 2.19, *p* = 0.14] nor externalizing problems [*R*^2^ = 0.002, *F*(1,449) = 1.02, *p* = 0.31] at the same time point (Supplementary Table S2).

### Longitudinal Changes in Internalizing and Externalizing Problems

To test the general effects of time on internalizing and externalizing behavior, *t*-tests were performed for both dependent measures. These analyses showed that parent-reported internalizing problems increased over time: internalizing problems at T2 (*M* = 3.22, SD = 2.81) were significantly higher than internalizing problems at T1 [*M* = 2.97, SD = 2.61, *t*(450) = −2.58, *p* = 0.01]. However, externalizing problems scores at T1 (*M* = 4.44, SD = 3.23) did not differ from externalizing scores at T2 [*M* = 4.60, SD = 3.34, *t*(450) = −1.63, *p* = 0.10].

### Longitudinal Predictions of Problem Behavior

To test our hypothesis that prosociality and reactive aggression may predict change in problem behavior over time, we then performed multiple regression analyses with prosociality, reactive aggression and problem behavior at T1 as predictors for problem behavior (either internalizing or externalizing) at T2. Subsequently, we repeated the analyses with prosociality^∗^reactive aggression and problem behavior at T1 as predictors. In line with prior research that focused on specific quadrants of bi-dimensional models, we did not include main effects in this analysis (Blankenstein et al., 2019). Regression coefficients are presented in Table 2.

**TABLE 2:** Regression coefficients of the four longitudinal analyses on problem behavior with prosociality, reactive aggression and the interaction variable as predictor.

	Internalizing T2			Internalizing T2			Externalizing T2			Externalizing T2		
	b	SE	β	b	SE	β	b	SE	β	b	SE	β
Constant	3.23**	0.10	–	3.23**	0.10	–	4.61**	0.09	–	4.61**	0.09	–
Internalizing T1	1.95**	0.12	0.69	1.95**	0.12	0.69	–	–	–	–	–	–
Externalizing T1	–	–	–	–	–	–	2.66**	0.09	0.80	2.66**	0.09	0.80
Prosociality	–0.07	0.10	–0.03	–	–	–	–0.19	0.10	–0.06	–	–	–
Reactive aggression	0.04	0.10	0.01	–	–	–	–0.15	0.10	–0.05	–	–	–
Prosociality*reactive aggression	–	–	–	0.00	0.11	0.00	–	–	–	−0.24*	0.11	–0.07

Heteroscedasticity-consistent standard error estimates. *p < 0.029 (α Bonferroni-corrected for correlated variables), **p < 0.001.

#### Prediction of Internalizing Behaviors

A multiple regression analysis for internalizing problems showed that, as expected, internalizing problems at T2 were positively predicted by internalizing problems at T1 [*R*^2^ = 0.48, *F*(3,447) = 86.08, *p* < 0.001, *b* = 1.95, SE = 0.12, β = 0.69], but not by prosociality (*b* = −0.07, SE = 0.10, β = −0.03, *p* = 0.47) or reactive aggression (*b* = 0.04, SE = 0.10, β = 0.01, *p* = 0.71). A separate regression analysis showed that prosociality^∗^reactive aggression also did not explain additional variance above internalizing problems at T1 (*b* = 0.00, SE = 0.11, β = 0.00, *p* = 0.99).

#### Prediction of Externalizing Behaviors

**FIGURE 3:**
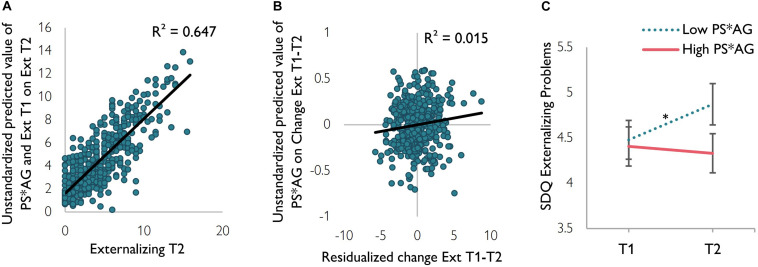
**(A)** Model fit of the regression with externalizing problems at T1 (Ext T1) and the prosociality*aggression interaction (PS*AG) as predictors for externalizing problems at T2 (Ext T2). **(B)** Model fit of the regression with the prosociality*aggression interaction as predictor for the change in externalizing problems from T1 to T2 (Change Ext T1–T2). The *x*-axis displays the unstandardized residuals of externalizing problems at T1 regressed on externalizing problems at T2. **(C)** Visualization of the longitudinal development of externalizing problems for low and high scores on interaction of prosociality (PS) and reactive aggression (AG). Groups were based on median split of the interaction variable of SNAT and PCG on time point 1. Error bars represent standard errors. T1, time point 1; T2, time point 2.

A multiple regression analysis for externalizing problem behavior showed that, as expected, externalizing problems at T2 were best predicted by externalizing problems at T1 [*R*^2^ = 0.65, *F*(3,447) = 313.20, *p* < 0.001, *b* = 2.66, SE = 0.09, β = 0.80, *p* < 0.001]. Prosociality showed a negative association with externalizing problems at T2 (*b* = −0.19, SE = 0.10, β = −0.06, *p* = 0.06), although this failed to reach significance. Reactive aggression did not predict externalizing problems at T2 (*b* = −0.15, SE = 0.10, β = −0.05, *p* = 0.12). Most importantly, a separate regression analysis showed that prosociality^∗^reactive aggression negatively predicted externalizing problems at T2 (*b* = −0.24, SE = 0.11, β = −0.07, *p* = 0.027), in addition to the initial level of externalizing problems at T1 (*R*^2^ total model = 0.65, Figure 3A, *R*^2^ prosociality^∗^reactive aggression = 0.015, Figure 3B). To further investigate this effect, we divided participants into two groups of either high or low prosociality^∗^reactive aggression, based on median split. *Post hoc* paired sample *t*-tests showed that the effect was mainly driven by low interaction scores (Figure 3C): children who scored low on prosociality^∗^reactive aggression (low prosociality and low reactive aggression) showed an increase in externalizing problems between T1 [*M* = 4.48, SD = 3.23) and T2 (*M* = 4.87, SD = 3.42, *t*(225) = −2.86, *p* = 0.005]. Children who scored high on prosociality^∗^reactive aggression (high prosociality and high reactive aggression) did not significantly differ in externalizing problems between T1 (*M* = 4.41, SD = 3.23) and T2 [*M* = 4.33, SD = 3.24, *t*(224) = 0.55, *p* = 0.58].

#### Robustness Checks

To investigate the longitudinal effect on externalizing problems further, we tested whether excluding the participants (*n* = 126) that only had SDQ scores of one parent affected the results. The results did not change: prosociality was still negatively related to externalizing problems at T2, although non-significant (*b* = −0.21, SE = 0.11, β = −0.07, *p* = 0.06, controlled for externalizing problems T1 and reactive aggression). Prosociality^∗^reactive aggression remained a negative predictor of externalizing problems at T2, controlled for externalizing problems at T1 (*b* = −0.22, SE = 0.11, β = −0.07, *p* = 0.045).

Additionally, since prior research revealed effects of age, gender, and SES on externalizing problems (Bongers et al., 2004; Leve et al., 2005; Silver et al., 2005), we checked for the effects of those three variables using stepwise regression analyses. Only gender was a significant predictor of externalizing problems at T2, controlled for externalizing problems at T1 (gender: *b* = −0.52, SE = 0.19, β = −0.08, *p* = 0.007), indicating that boys showed a higher increase in externalizing problems at T2 than girls.

Finally, we tested whether the effects remained significant when controlling for gender. Controlled for gender, prosociality still showed a negative association with externalizing problems at T2 that, however, failed to reach significance (*b* = −0.19, SE = 0.10, β = −0.06, *p* = 0.07, controlled for reactive aggression, externalizing problems at T1 and gender). However, prosociality^∗^reactive aggression remained a significant predictor of externalizing problems at T2 (*b* = −0.24, SE = 0.11, β = −0.07, *p* = 0.025, controlled for externalizing problems at T1 and gender).

## Discussion

The present study investigated whether a bi-dimensional perspective on prosociality and reactive aggression predicted problem behavior, both cross-sectionally and longitudinally. Prosociality and reactive aggression were not correlated, which is consistent with prior research suggesting that these are separable constructs (Pulkkinen, 1984). Even though there were no relations with problem behavior cross-sectionally, the interaction of prosociality and reactive aggression was predictive of externalizing problems over time. Specifically, children who scored low on both prosociality and reactive aggression (i.e., the passive bystanders) showed an increase in externalizing problems 1 year later, in contrast to children who scored high on both constructs (i.e., the strong responders to social environment). These findings fit with recent studies showing that bi-directional models seem to have additional value in predicting developmental outcomes, such as problem behavior (Sunami et al., 2019).

In previous research, prosocial, and antisocial behaviors were often regarded as opposing constructs, but the lack of correlation between prosociality and reactive aggression in our study adds to the idea that prosocial and aggressive behaviors are independent characteristics within an individual (Pulkkinen, 1984). This idea is further supported by findings of relatively independent trajectories of prosocial behavior and physical aggression in middle childhood (Kokko et al., 2006), and differential genetic and environmental mechanisms underlying altruism and antisocial behaviors (Krueger et al., 2001). Interestingly, variability between individuals in the relation between prosociality and reactive aggression were predictive of externalizing problems over time.

The additive effects of prosociality and reactive aggression negatively predicted the change in externalizing problems across 1 year, indicating reduced externalizing behavior for the “strong responders to social environment” compared to the “passive bystanders” in our model. This predictive effect of the interaction term supports the hypothesis that it might not necessarily be detrimental for an individual to show aggression when combined with prosocial behavior, which supports prior research showing that adolescents who use prosocial and proactive aggressive strategies are well-adjusted and popular among peers (Hawley, 2003, 2014; Hartl et al., 2019). These high social positions within the peer group seem to be related to adaptive interactions with others (Allen et al., 2005) and aggression combined with popularity might aid as buffer against social problems (Rose and Swenson, 2009). Additionally, both reactive aggression and prosociality possibly aid in maintaining and fostering positive self-views. As reactive aggression might result from threats to self-evaluations (Yoon et al., 2018), defending oneself could be a way to protect against negative self-views. Prosociality might result in self-enhancement (Crone and Fuligni, 2020). Positive self-concept is positively related to social adjustment factors and can protect against problem behavior in adolescence (Ybrandt, 2008; Lee and Stone, 2012). Therefore, an interesting approach for future studies is to focus on the mediating role of self-evaluations following reactive aggressive and self-enhancing prosocial behavior, and the subsequent relation to behavioral adjustment.

However, the strongest effect on externalizing problems was found in the group that scored low on the interaction term of prosociality and reactive aggression. Showing neither prosocial compensating behavior nor reactive aggression was associated with an increase in externalizing problems over time. Adolescents who did not use prosocial or aggressive strategies (“non-controllers” or “passive bystanders” in our model) were previously found to be among the most peer rejected, even more so than adolescents who only used one strategy (Hawley, 2003). Peer rejections and victimization in childhood have often been related to adjustment difficulties and externalizing and internalizing problems (Cillessen and Lansu, 2015). These results seem to suggest that social experiences within the peer group might also be important in explaining adjustment problems. Yoon et al. (2018) showed that reactive behavior in youth are mostly based on immediate negative social evaluations (trial to trial), whereas reactive bias based on accumulated negative experiences (over several trials) is more prevalent in adults. An interesting direction for future research is to incorporate the social position within a peer group as an additional factor, to test whether and how peer experiences can explain additional variance in the relation between social competence and developmental outcomes.

Previous studies specifically investigated the combination of goal-directed or proactive aggression and prosociality, whereas our results extend these findings by showing that the effects also hold for prosociality and reactive aggression. Although reactive and proactive aggression are strongly correlated (Card and Little, 2006), differential patterns of developmental outcomes have been associated with each type of aggression. Several studies report more negative psychosocial outcomes for reactive aggression than for proactive aggression (Card and Little, 2006; Mcauliffe et al., 2006). However, the definition of reactive aggression differs across studies, as it is often focused on the tendency to over-attribute hostility to others (Dodge and Coie, 1987). When reactive aggression is investigated in the light of self-defense, i.e., standing up for oneself, as was the case in our study, it seems to have more favorable developmental outcomes, such as less internalizing and externalizing problems, compared to proactive aggression (Pulkkinen, 1996). This interpretation is consistent with the current findings showing that being aggressive to protect yourself is negatively predictive of externalizing problems when it co-occurs with prosocial behavior.

There were also findings that were not consistent with the hypotheses. Contrary to our expectation, levels of internalizing problems were not predicted by prosociality, reactive aggression or its interaction term. A possible explanation is that the two experimental tasks we used focused mainly on active behavior following social feedback, i.e., aggression and prosociality, but did not explicitly test emotional states following social feedback. This active behavior might relate more to externalizing than to internalizing behaviors. Furthermore, parental report might not be the most suitable approach to measure internalizing problems, since these behaviors are not always observable from the outside and therefore more difficult for the parent to report (Youngstrom et al., 2000). It should also be noted that even though the SDQ is a widely used screening instrument for psychopathology, it is not the most sensitive measure to capture the full range of behavioral problems. Therefore, we encourage future studies to use a more thorough (self-report) questionnaire or diagnostic interview to measure problem behavior. Nevertheless, the development of internalizing behavior in our study was consistent with prior research, as reported levels of internalizing problems increased over time (Leve et al., 2005). These findings reassure that the study sample is generalizable to other studies. For externalizing problems, we did not observe a change across 1 year in middle childhood. Although prior studies focusing on a longer period of development reported a decrease in externalizing problems over time (Bongers et al., 2004; Leve et al., 2005), it should be noted there are individual differences in these trajectories (e.g., Kokko et al., 2006). Also, externalizing problems might seem more stable when investigated in only a 1-year period (see for example, Mcauliffe et al., 2006). Finally, boys showed higher levels of externalizing problems compared to girls, which is a consistent finding in the literature (Broidy et al., 2003; Bongers et al., 2004).

Thus, our study has several strengths. This study is the first to investigate the co-occurrence of prosociality and reactive aggression and associated adjustment outcomes in a unique large longitudinal sample. The effect of prosociality and reactive aggression on externalizing problems over time was robust even after correcting for multiple possible confounders. The use of well-validated experimental tasks eliminated reporter effects that often occur in questionnaire data (Veenstra et al., 2008). In addition, the use of multi-informant SDQ data provides a richer assessment of children’s problems compared to reports of one parent only. Furthermore, the SNAT specifically focused on aggression following social feedback, therefore it specifically measured reactive aggression, reducing previously reported difficulties to disentangle reactive and proactive aggression (Card and Little, 2006). Since participants received feedback from unknown peers they would not meet in real life, it is unlikely that their aggression was proactive or goal-directed. So, it seems the primary function of aggression as measured with the SNAT was to release frustration following negative social feedback and to maintain positive self-evaluations.

However, some limitations should be considered as well. First, the effects found in our study were small and therefore need replication. Although experimental tasks are useful in measuring a construct in a specific context or state, they might not always generalize to other situations, which might be a possible explanation for the small effects. Especially prosocial behaviors are very diverse and can be methodologically challenging to capture (El Mallah, 2020). Therefore, we cannot exclude the possibility that our findings are task-specific. Future research should tackle this issue by testing the bi-dimensional taxonomy in other social contexts, such as in situations that are more costly, or by using a combination with more trait-like measures, such as multi-informant questionnaire data. Second, scores and variance in externalizing and internalizing problems levels were relatively low, as might be expected in a population sample. An interesting direction for future research is to test whether the proposed bi-dimensional taxonomy can also be used to explain individual differences in a clinical population where externalizing and internalizing problems and social adaptation problems are more common (e.g., Boonen et al., 2014). Furthermore, we measured prosociality and reactive aggression on a continuous scale as our aim was to test for relations between the two constructs and in this way we could optimally use the variation in prosociality and reactive aggression. However, we did not group participants into one of four subtypes as described in Crone et al. (2020). Using a more data driven approach in future research might help identifying these four subtypes in the population. Finally, we defined reactive aggression as the tendency to show aggressive behavior when there is threat to self-evaluations, such as when receiving negative feedback, compared to when there is no need for self-defense, i.e., when receiving positive feedback. Difference scores can be influenced by multiple factors and therefore replication across tasks is needed.

## Conclusion

In conclusion, our results suggest that reactive aggression and prosociality are separable constructs (Pulkkinen, 1984). Moreover, we showed that aggression is not necessarily maladaptive at the individual level when it has a self-protective function and when it is combined with prosocial behavior (Hawley, 2003; Hartl et al., 2019). Specifically, this combination of prosociality and reactive aggression could indicate social responsivity and behavioral adaptation to changes in the social environment. Although we stress the need for replication of our results, the finding that aggression does not necessarily have maladaptive effects for the individual might have implications for interventions that focus on minimizing aggression (Farmer and Xie, 2007). Furthermore, our findings underscore the importance of studying social competence across multiple dimensions, as externalizing problems only arose when combining constructs of prosociality and reactive aggression. Using bi-dimensional taxonomies could be a way forward in our understanding of the interrelations between complex social behaviors, which could ultimately help children succeed in their social life.

## Data Availability Statement

The original contributions presented in the study are available on request. This data can be found on Dataverse NL: https://doi.org/10.34894/WH8KOG.

## Ethics Statement

The studies involving human participants were reviewed and approved by the Dutch Central Committee on Research Involving Human Subjects (CCMO). Written informed consent to participate in this study was provided by the participants’ legal guardian/next of kin.

## Author Contributions

SD and EC drafted the manuscript. MA and MM collected the data. SD analyzed the data with assistance from AD, MA, MM, and EC. All authors contributed to the design and final manuscript.

## Conflict of Interest

The authors declare that the research was conducted in the absence of any commercial or financial relationships that could be construed as a potential conflict of interest.

## Footnotes

1 http://www.sdqinfo.org

2 https://www.quantitativeskills.com/sisa/calculations/bonfer.htm
