# Supplementary material for: A Bi-Dimensional Taxonomy of Social Responsivity in Middle Childhood: Prosociality and Reactive Aggression Predict Externalizing Behavior Over Time
Source: Front Psychol. 2021 Jan 15;11:586633. doi: 10.3389/fpsyg.2020.586633 (PMC7843792; doi:10.3389/fpsyg.2020.586633)
Supplement: Supplementary file 1 [file Table_1.DOCX]

**Supplementary materials**

|  | PCG Δ exclusion – inclusion | | | PCG exclusion | | PCG inclusion | |
| --- | --- | --- | --- | --- | --- | --- | --- |
|  | | *r* | *p* | *r* | *p* | *r* | *p* |
| SNAT Δ negative – positive | | .05 | .34 | .04 | .34 | -.01 | .91 |
| SNAT negative | | .00 | .93 | .04 | .33 | .07 | .12 |
| SNAT positive | | -.07 | .17 | -.02 | .61 | .07 | .16 |

**Table S1.** Correlations between prosocial variables (PCG) and reactive aggression variables (SNAT) at T1.

*Note.*  P-values were corrected with heteroscedasticity-consistent standard error estimators.

**Table S2.** Regression coefficients of the cross-sectional regressions on problem behavior with prosociality, reactive aggression and the interaction variable as predictors.

|  | Internalizing T1 | | | Internalizing T1 | | | Externalizing T1 | | | Externalizing T1 | | |
| --- | --- | --- | --- | --- | --- | --- | --- | --- | --- | --- | --- | --- |
|  | b | SE | β | b | SE | β | b | SE | β | b | SE | β |
| Constant | 2.97* | .12 | - | 2.97* | .12 | - | 4.44* | .15 | - | 4.44* | .15 | - |
| Prosociality | .12 | .12 | .05 | - | - | - | -.06 | .15 | -.02 | - | - | - |
| Reactive aggression | .12 | .12 | .05 | - | - | - | -.19 | .15 | -.06 | - | - | - |
| Prosociality * Reactive aggression | - | - | - | .19 | .13 | .07 | - | - | - | -.16 | .15 | -.05 |

*Note.*  Heteroscedasticity-consistent standard error estimates. **p* < .001.
